# Natural Compounds for Controlling Plant Pathogens

**DOI:** 10.3390/plants15071084

**Published:** 2026-04-01

**Authors:** José Sebastián Dambolena, Virginia Lara Usseglio

**Affiliations:** Instituto Multidisciplinario de Biología Vegetal (IMBIV-CONICET), Facultad de Ciencias Exactas, Físicas y Naturales, Universidad Nacional de Córdoba, Córdoba X5000HUA, Argentina; virginia.usseglio@unc.edu.ar

Agricultural production systems across the world are currently facing unprecedented challenges. Rapid environmental changes, increasing climatic variability, and the growing demand for food production have intensified the pressure on agroecosystems [1,2]. Among the most important factors limiting crop productivity are plant diseases caused by fungi, insects, and bacteria. These pathogens can lead to substantial yield losses, deterioration in crop quality, and significant economic damage, representing potentially serious risks for food safety and human health via toxin production [3,4]. Consequently, the effective management of plant pathogens remains a central priority for both agricultural research and global food security.

Pest management has traditionally relied on a combination of strategies that include developing resistant crop varieties and applying synthetic pesticides, among others. Over the past decades, these approaches have played a crucial role in protecting crops and maintaining agricultural productivity. However, the extensive application and misuse of these methods have raised increasing concerns regarding their environmental and ecological impacts. Residues of synthetic agrochemicals may accumulate in soil and water systems, negatively affecting non-target organisms and contributing to biodiversity loss. Moreover, the continuous exposure of pathogens to chemical treatments often accelerates the development of resistance, thereby reducing the long-term effectiveness of these strategies [5,6,7,8,9,10].

In response to these challenges, developing environmentally sustainable alternatives has become an important focus of research in crop protection. Among the most promising approaches is the use of natural compounds derived from plants, microorganisms, and other biological sources. Natural products represent extraordinarily diverse chemical groups, including phenolics, terpenoids, alkaloids, and flavonoids, many of which exhibit strong pesticidal properties. Compared with synthetic pesticides, natural compounds often present several advantages that make them attractive candidates for sustainable crop protection. Many of these substances are biodegradable and tend to have lower environmental persistence, thereby reducing the risk of long-term environmental contamination. In addition, they frequently display lower toxicity toward non-target organisms and may present improved safety profiles for both agricultural workers and consumers. Another important advantage lies in their structural diversity and complex modes of action, which may reduce the likelihood of pests developing resistance [11,12,13,14,15,16].

This Special Issue *“Natural Compounds for Controlling Plant Pathogens”* was organized with the aim of bringing together recent advances in this dynamic and interdisciplinary field. The collection brings together original research articles that address different aspects of natural-product-based plant disease management, including discovering new bioactive substances, evaluating their activity against important phytopathogens, and exploring their potential mechanisms of action.

The nine articles included in this volume illustrate the diversity of natural sources and research approaches currently being explored for plant pathogen control. These are as follows:

- “Paralysis Activity of “Basic Substances” and Rose Extracts on *Meloidogyne incognita* Second-Stage Juveniles”
- “Chemical Composition and Antifungal Activity of *Artemisia sieversiana* Essential Oil Growing in Jilin Against Black Spot on Yanbian Pingguoli Pear in China”
- “Organic Compounds as a Natural Alternative for Pest Control: How Will Climate Change Affect Their Effectiveness?”
- “Antimicrobial Potential of Six Plant Essential Oils Against *Pseudomonas syringae* pv. actinidiae: In Vitro Activity and In Planta Efficacy Do Not Always Align”
- “In Vitro Evaluation of the Antifungal Activity of *Trigonella foenum*-*graecum* Seed Extract and Its Potential Application in Plant Protection”
- “Antifungal Activity of *Artemisia capillaris* Essential Oil Against Alternaria Species Causing Black Spot on Yanbian Pingguoli Pear in China”
- “Eco-Friendly Crop Protection: *Argyrantemum frutescens*, a Source of Biofungicides”
- “Bioactive Sesquiterpenoids from *Santolina chamaecyparissus* L. Flowers: Chemical Profiling and Antifungal Activity Against Neocosmospora Species”
- “Antifungal Activity of Genistein Against Phytopathogenic Fungi *Valsa mali* Through ROS-Mediated Lipid Peroxidation”

Taken together, the contributions in this volume illustrate the diversity of natural compounds with potential applications in crop protection, highlighting their relevance in developing environmentally friendly strategies. The collective contributions of this Special Issue therefore represent an important step toward advancing knowledge in the field of natural-product-based plant protection. By highlighting both fundamental research and potential practical applications, the articles included in this volume contribute to the development of innovative approaches aimed at improving plant health, reducing environmental impacts, and promoting more sustainable agricultural systems.

Finally, we would like to express our sincere gratitude to all the authors who contributed their valuable research to this Special Issue. Their work reflects the growing global effort to develop environmentally sustainable solutions for plant disease management. We hope that the research presented in this volume will inspire further studies and foster new collaborations aimed at advancing the use of natural compounds in sustainable crop protection.
